# Vagifem is superior to vaginal Premarin in induction of endometrial thickness in the frozen-thawed cycle patients with refractory endometria: A randomized clinical trial

**Published:** 2014-06

**Authors:** Jaleh Zolghadri, Hossein Haghbin, Nasrin Dadras, Shabnam Behdin

**Affiliations:** 1 *Infertility Research Center, Shiraz University of Medical Sciences, Shiraz, Iran.*; 2 *Department of Obstetrics and Gynecology, Shiraz University of Medical Sciences, Shiraz, Iran.*

**Keywords:** *Premarin*, *Estradiol*, *Endometrium*, *Infertility*, *Human*

## Abstract

**Background:** Embryo transfer to a developed endometrium is an important prognostic factor in frozen-thawed embryo transfer cycle outcome. Vaginal estrogen, such as Vagifem vaginal tablets and Premarin vaginal cream, is a regimen used for the patients with refractive endometria.

**Objective: **Our objective was to compare the effects of Vagifem and Premarin on the endometrial thickness of the patients with refractive endometria.

**Materials and Methods: **In this randomized clinical trial, 30 patients with refractive endometria in frozen-thawed embryo transfer cycles received Vagifem vaginal tablets and 30 women received Premarin vaginal cream. Endometrial thickness was measured on the 14th day of drug administration.

**Results: **Comparing the endometrial thicknesses of the two groups showed that the endometria of the Vagifem group was significantly thicker than that of the Premarin group (5.93±0.38 vs. 6.74±0.32; p<0.001).

**Conclusion:** Vagifem is superior to Premarin in induction of endometrial thickness in frozen-thawed embryo transfer cycles in the patients with refractive endometria.

## Introduction

Success of embryo transfer, one of the most important steps of assisted reproductive technology (ART), depends on good quality embryo, receptive endometrium, and an appropriate interaction amongst them (1, 2). Endometrium must be mature to become receptive to the functional embryo. Follicular and luteal phases are stages of a natural ovulatory cycle that, by controlling the right amount of body estrogen (E_2_) and progesterone (P), lead to proliferation of the endometrial cells and induction of endometrial receptivity (3).

In 1983, a great achievement was gained in the field of ART when the endometrium of a woman with ovarian failure was made receptive by the means of exogenous E_2_ and P administration (4). Since then, there have been many developments in this field. Studies have shown that E_2_ and P are sufficient for making an endometrium receptive in the women with no function of ovaries (5). Yet, routes, dosages, and duration of administration of these drugs are the variables that have been subject to many revisions and improvements and no evidence is available regarding the superiority of one method to another (6). In general, E_2_ can be administered through oral, intra-muscular, vaginal, and transdermal routes. Oral route is simple, easy, and effective. Transdermal route, which is used when the oral route is not effective, leads to more Endometrial Thickness (ET) but with the same pregnancy rates compared to the oral route (6, 7).

Endometrium development is controlled by serial ultrasonography in artificial cycles (8). A positive correlation has been found between ET and pregnancy rate in In Vitro Fertilization (IVF) and embryo transfer patients. Moreover, other studies have reported adverse effects of excessively increased ET on decreasing the pregnancy rate (9-13). In contrast, some studies failed to find a relationship between ET and pregnancy rate (14-17). Furthermore, Remohi *et al* revealed an association between ET and implantation rate, underscoring the significance of ET in embryo transfer cycles (18). Nevertheless, not all the patients’ endometria respond to programmed sequential treatment with exogenous E_2_ and P. 

Up to now, many techniques, including aspirin, sildenafil, pentoxifylline-vitamin E, repeated endometrial biopsies, and prolonging the duration of E_2_ therapy, have been developed to improve ET in cases with poorly responsive endometria (19-23). Nonetheless, many of these therapies are far from perfect, and controversies exist regarding their effectiveness. Vaginal E_2_ is yet another method for endometrial receptivity improvement (23). Not only does this route result in high E_2_ serum levels, but it also causes a higher level in the endometrium; hence, it is used in refractory cases (24). 

Premarin and Vagifem are examples of vaginal E_2_ regimens. Premarin or conjugated equine estrogen (CEE) is a naturally occurring E_2_ derived from urine of mares. Conjugation enables the compound to be water-soluble and more absorbable (25). Premarin is also the third most common drug used in the U.S. (26). Vagifem is a vaginally delivered Ethinyl Estradiol tablet. Estradiols are the most commonly used synthetic estrogens (25). Besides, Vagifem has the advantage of being superior to other vaginal estrogens in the Hormone Replacement Therapy (HRT) patients' point of view (27, 28). Considering the controversy regarding the use of a therapy for the embryo transfer patients with refractory endometria to the initial therapies, we decided to study the effects of these two therapies, namely Vagifem and vaginal Premarin, on ET development.

## Materials and methods

**Figure 1:**
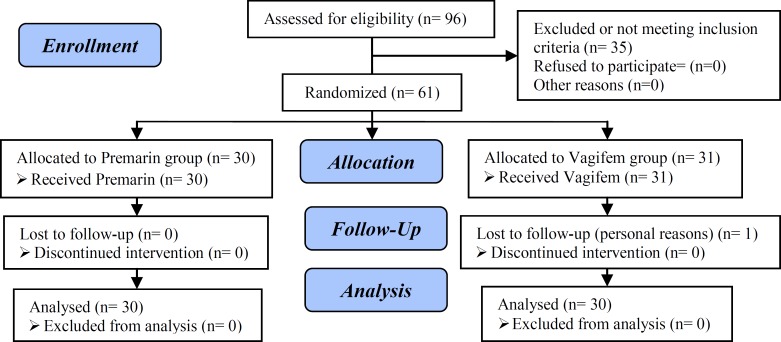
Consort flow chart of the progress of participants through each state of the trial

The present randomized clinical trial (RCT) was conducted on the women with failure to achieve optimal ET (<3 mm) in frozen-thawed embryo transfer cycles by conventional estrogen therapy. In IVF cycles, some embryos are cryopreserved for later use in the subsequent frozen-thawed embryo transfer cycles. Even increasing the estrogen dosage or changing the type of estrogen to Ethinyl Estradiol was not effective in these women (29). Written informed consents were obtained from all the study patients before recruitment and the study was approved by the Ethics Committee of Shiraz University of Medical Sciences, Shiraz, Iran. Consort flow diagram of the study is demonstrated in figure 1.

Using computer generated block randomization method, 61 women in the reproductive age were sampled from the infertile patients aging 24-42 years who were unresponsive to conventional and added E_2_ regimens of embryo transfer cycles in Ghadir Hospital, Shiraz, Iran, a tertiary referral center, in 2013. Sample size was estimated by calculation of variance of ET of similar patients in our previous pilot studies. The exclusion criteria of the study were having a previous medical history of tuberculosis, Asherman syndrome, endometriosis, uterine leiomyoma, pelvic inflammatory diseases, collagen vascular diseases, and other diseases that secondarily hinder pregnancy. Smokers and alcohol abusers were also excluded from this study. Patients with undiagnosed Abnormal Uterine Bleeding (AUB), history of thromboembolic diseases, E_2_ dependent malignant neoplasms, and liver disease were also excluded from this study due to contraindications to E_2_ (30). All the patients underwent pelvic ultrasonography one month prior to the study to rule out any polyps or anatomical problems.

The patients were randomly divided into two groups. The patients in group 1 (n=30) received 1.25 mg/day (half of vaginal applicator) Premarin in the form of vaginal cream (Estromarin vaginal cream, 2.5 mg, Aburaihan pharmaceutical, Iran). On the other hand, those in group 2 (n=31) received 20 micrograms Vagifem (1 tablet Bid) (vaginal tablet, 10 mcg, Novo Nordisk, Denmark). One of the volunteers in group 2 exited the study in follow-up. It should be mentioned that both regimens were added to the conventional E_2_ regimen. Due to the fact that Vagifem was administered in the form of vaginal tablets and Premarin in the form of vaginal cream, blinding was impossible on the side of the patients. 

From the second day of the menstrual cycle, the patients received 8 mg or 4 oral tablets of Estradiol Valerate (Progynova, 2mg tablets, Schering, Germany) daily. Vagifem and Premarin were added from the third day of the menstrual cycle for 14 days. Ultrasonographic measurement of ET was performed in both groups. The ET was measured in this period and at the end of the 14^th^ day of Vagifem or Premarin administration, which equals to the 16^th^ day of the menstrual cycle, and was compared between the two groups. 

From the 16^th^ day of the menstrual cycle, 400mg per day of vaginal P suppositories (Cyclogest vaginal suppository 200 mg: Shire, Andover, Hants, UK) was administered. Finally, on the 19^th^ day of the menstrual cycle, frozen-thawed embryos were transferred to our patients by an expert obstetrician. After confirmation of an adequately full bladder, the uterine cavity was found through ultrasound. Then, the embryos were transferred in the lithotomy position and were deposited at 1.5 cm below the uterine fundus. The catheter was withdrawn gently, while maintaining the pressure on the syringe plunger.

**Statistical analysis**

In this study, ET was compared between the two groups using Student *t*- test. Besides, p-values less than 0.05 were considered as statistically significant.

## Results

**Figure 2:**
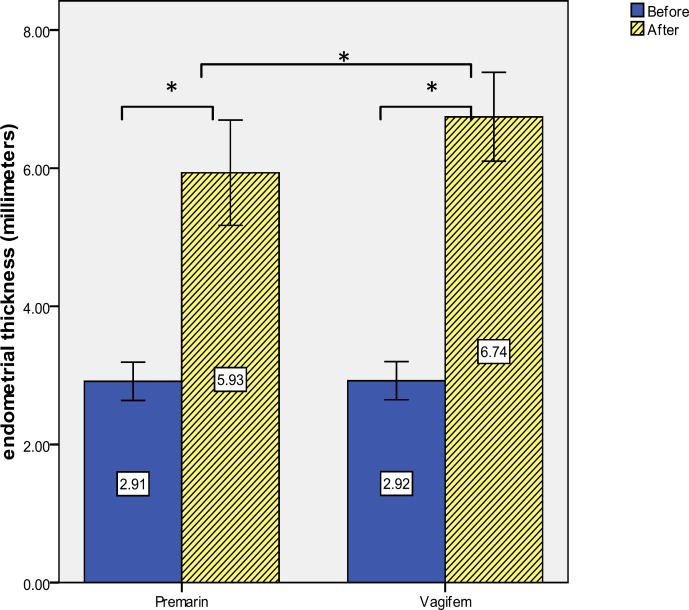
Endometrial thicknesses (millimeters) before and after the therapy with Premarin and Vagifem.

In this RCT, 2 groups of Premarin and Vagifem were compared in terms of ET. Within-group comparisons were also made in the subjects of each group before and after the therapy. Table I shows some possible confounding factors, including age, Body Mass Index (BMI), and the duration of infertility in the Premarin and Vagifem groups. As the table depicts, no significant difference was found between the two groups regarding these factors. As figure 2 shows, the initial ET was also the same between the two groups. According to figure 2, ET in the Premarin group ranged from 5.1-6.7 mm, with a mean±SD of 5.93±0.38 millimeters. On the other hand, this measure ranged from 6-7.5 mm, with a mean±SD of 6.74±0.32 mm in the Vagifem group. 

According to the results of student *t*-test, both measures significantly out-weigh their initial values which were less than 3 millimeters (p<0.001). Furthermore, comparison of the two groups' ETs showed that the ET of the Vagifem group was significantly higher compared to that of the Premarin group (5.93±0.38 vs. 6.74±0.32; p<0.001). In addition, the mean difference (±c) was 0.81±0.09.

**Table I:** Age, BMI, and duration of infertility in Premarin and Vagifem groups

	**Premarin group**	**Vagifem group**	**p-value** *
Age (years)	31.93 ± 4.30	33.06 ± 3.08	0.248
BMI (kg/m^2^)	23.26 ± 1.24	23.76 ± 1.47	0.171
Duration of infertility (years)	6.76 ± 1.59	6.26 ± 1.38	0.200

Number are presented as mean±SD. Student t-test was used for comparison.

* Not significant.

*p<0.001. Error bars: +/- 2 SD bars: +/- 2 SD

## Discussion

Although some studies have failed to find a relationship between ET and pregnancy rate, most experts agree that embryo transfer to endometria with >7mm thickness is a good prognostic indicator of pregnancy after embryo transfer (9-17). Up to now, different E_2_ and P regimen protocols with various routes of administration have been introduced (6). Other formulations, such as aspirin, and methods, such as repeated endometrial biopsies, have also been suggested. However, none of these has been proven to be superior to others (6, 19, 22). 

Considering the fact that Vagifem and Premarin are widely used medications and controversial evidence exists regarding the superiority of a regimen over the other for the patients with refractory endometria, we decided to compare these two medications. Up to now, no studies have compared the role of Vagifem and Premarin in ET development in IVF and embryo transfer patients. The findings of the present study revealed the superiority of Vagifem to Premarin in ET improvement. Vagifem is also more acceptable in the HRT patients’ point of view (27). 

In this study, we focused on ET, while there are more variables playing part in successfulness of frozen-thawed embryo transfer cycles. In a comprehensive retrospective study on 2450 patients, Cai *et al* concluded that out of the 27 candidate variables for prognosis of a good IVF and embryo transfer cycle outcome, 9 had the highest correlation with success in IVF and embryo transfer cycles (31). These variables included age, quality and quantity of embryos, ET, and duration of infertility. Out of these factors, the total number of good-quality embryos had the highest correlation with successful outcomes, even higher than ET. This can explain the reason for lower success rate when the number of the good-quality embryos dropped significantly in the women older than 35 years (32). 

Success also depends on other factors, such as endometrial texture and sub-endometrial blood flow. In a study on 1933 women, Zhao *et al* came to this conclusion that in an endometrium with an adequate thickness of 7-14 mm, a triple-line pattern conferred the best clinical outcome (32). Singh *et al* also ruled in sub-endometrial blood flow as another prognostic factor (33). Although ultra-low-dose vaginal E_2_ is considered not effective on endometrium and, therefore, safe for HRT in postmenopausal women, proper vaginal E_2_ doses are more effective than oral E_2_ and recommended for use in embryo transfer patients over oral E_2_ (24). 

The patients who have had a refractory endometrium to conventional oral E_2_ are even more suitable for vaginal route (23). Serum and Endometrial E_2_ concentration is significantly higher in vaginal route compared to oral route (23, 34). Fanchin *et al* compared oral and vaginal route by monitoring ET and uterine perfusion after 14 days of E_2_ administration in 39 infertile women. Their study proved that vaginal route leads to a superior ET and uterine perfusion compared to oral route (35). The aforementioned studies depict that vaginal E_2_s are the best candidates for patients with refractive endometria. While Premarin and Vagifem are the most commonly used and studied vaginal estrogens for HRT, their use for embryo transfer patients is significantly less studied. This study has researched this important and novel area.

In spite of the fact that this study showed Vagifem to be superior to vaginal Premarin cream and previous studies have also revealed the superiority of Vagifem to Premarin from the HRT patients’ point of view. More studies with larger sample sizes are required to be conducted on these and further issues. For example, the pregnancy outcomes are needed to be compared in order to fully support Vagifem over Premarin.

## Footenote

***Registration ID in IRCT: IRCT2012103911306N1***

***This article extracted from M.D. thesis. (Hossein Haghbin)***
